# A Case Report of Purtscher-Like Retinopathy Secondary to *Pneumocystis jirovecii* Pneumonia in an Immunocompromised Patient

**DOI:** 10.1155/2022/7870179

**Published:** 2022-12-19

**Authors:** Alastair David Bezzina, Isaac Bertuello

**Affiliations:** Ophthalmology Department, Mater Dei General Teaching Hospital, Malta

## Abstract

To the best of our knowledge, we present the first case of a young adult male, on long-term immunosuppressive therapy following a live-donor kidney transplant, with Purtscher-like retinopathy (PLR) secondary to *Pneumocystis jirovecii* pneumonia (PCP). The patient had presented to a routine medical follow-up and complained of a few weeks' history of dyspnoea and tachycardia after minimal exertion followed by acute bilateral blurring of vision. Unfortunately, the patient did not respond to a trial of corticosteroid treatment and subsequently became severely visually impaired. This case report expands the aetiological spectrum behind PLR and helps portray the disease process which, in some cases, leads to severe ischaemic sequelae.

## 1. Introduction

PLR is an ischaemic retinopathy which occurs in the context of conditions other than trauma, which has been classically related to Purtscher's retinopathy (PR). PLR is thought to arise secondary to leucoaggregation in response to a variety of inflammatory aetiologies leading to arteriolar blockage and subsequent retinal ischaemia. As with other types of ocular vascular disorders, PLR presents with sudden loss of vision and on clinical examination one will observe cotton wool spots, intraretinal haemorrhages, and the pathognomonic “Purtscher flecken” which are areas of perivascular pallor representing inner retinal ischaemia [1]. Multiple aetiologies [2] have been associated with PLR in the literature but, to our knowledge, this case report describes the first case of PLR secondary to an opportunistic fungal infection in an immunocompromised patient.

## 2. Case Report

The patient in question is a 30-year-old Caucasian gentleman who was being followed up regularly by one of the local nephrology firms following a live-donor kidney transplant a few years back. The patient complained of a few weeks' history of dyspnoea and tachycardia on minimal exertion. He denied having other symptoms, but mild pitting lower limb oedema was noted on examination. The gentleman also complained of multiple patches of skin eruptions which had been slowly developing over the past 6 months.

His past medical and surgical history included chronic kidney disease secondary to meningococcal septicaemia at eight years of age. This progressed to end-stage chronic kidney disease at the age of twenty-one. He was first started on peritoneal dialysis and later converted to haemodialysis. At age twenty-eight, the patient received a live kidney transplant from his brother. Four months later, the transplanted kidney was biopsied after a rise in serum creatinine was noted. Histology showed mild tubulitis, suggesting early cell-mediated rejection, which led to the doses of his immunosuppressive medications to be increased.

At the time of presentation, the patient was on tacrolimus 3 mg twice daily, mycophenolate 720 mg twice daily, prednisolone 5 mg daily, omeprazole 20 mg daily, folic acid 5 mg daily, and bumetanide 0.5 mg daily. He had no known drug allergies. Family and social history were unremarkable.

Initial examination revealed a regular pulse rate of 95 beats per minute which increased to 120 beats per minute on walking, a blood pressure of 120/80 mmHg, and a SpO2 of 98% which remained stable on exertion. The patient was afebrile at the time of examination. Bilateral mild pitting oedema up to the ankles was present.

Initial investigations included laboratory tests which showed a normal neutrophil count of 8.30 (4.30–11.40 × 10^9^/L) with a lymphopenia of 0.74 (2.10–3.60 × 10^9^/L). Normocytic anaemia was present with a haemoglobin level of 11.0 g/dL (14.1–17.2 g/dL) and a haematocrit of 34.1% (40.4–50.4%). Platelet count was normal at 186 (146 − 302 × 10^9^/L). Creatinine was 120 *μ*mol/L (59–104 *μ*mol/L), which remained relatively stable since the renal transplant occurred, with a urea of 13.2 mmol/L (1.7–8.3 mmol/L) and an eGFR of 64 mL/min/1.73m^2^. C-reactive protein and erythrocyte sedimentation rate were slightly elevated at 14.4 mg/L (0–5 mg/L) and 14 mm 1st hr (8–12 mm 1st hr), respectively. Natriuretic peptide (NT-proBNP) level was also slightly elevated at 147 pg/mL (5–125 pg/mL). Liver function tests were normal. The serum albumin had decreased slightly when compared to previous results at 34 g/L (32–52 g/L), previously 48 g/L, however, still in the normal range. The serum tacrolimus level was 8.8 ng/mL which was in range. Serological tests for toxoplasma and leishmania and the autoimmune screen were all negative. A virology screen was carried out which included polymerase chain reaction (PCR) tests for herpes simplex, varicella zoster, cytomegalovirus, Epstein-Barr virus, BK virus, COVID-19, and other viral respiratory pathogens, all of which turned out negative. The lactate dehydrogenase level was normal but beta-D glucan was detected at >523 pg/mL (positive > 80 pg/mL). Urinalysis and urine microscopy, culture, and sensitivity were all normal. An electrocardiogram showed a normal sinus rhythm at 90 bpm, and an echocardiogram excluded any cardiac pathology. Chest X-ray was unremarkable.

**Figure 1:**
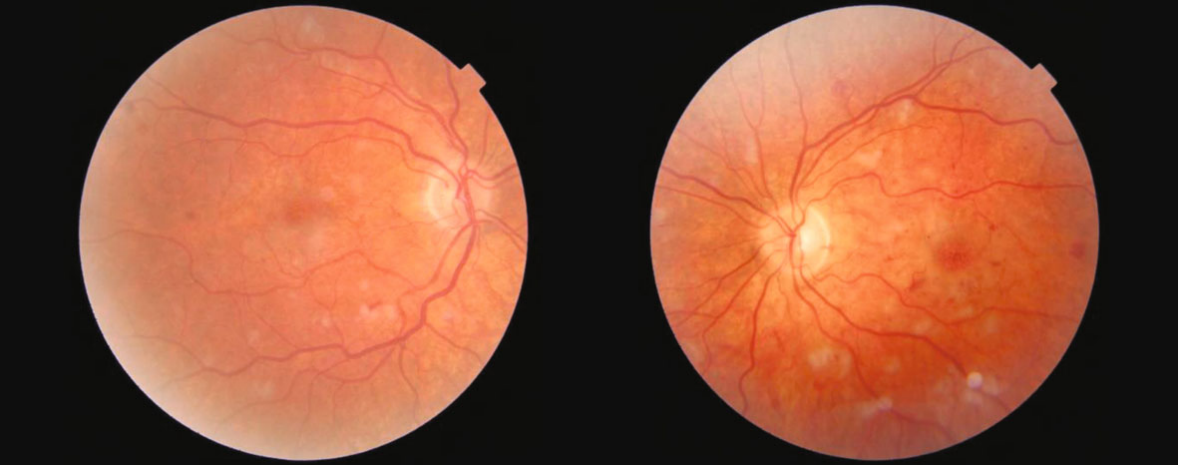
Fundal photos of the right and left eyes on presentation demonstrating intraretinal haemorrhages, cotton wool spots and Purtscher flecken with the characteristic clear zone between the lesions and the blood vessels.

**Figure 2:**
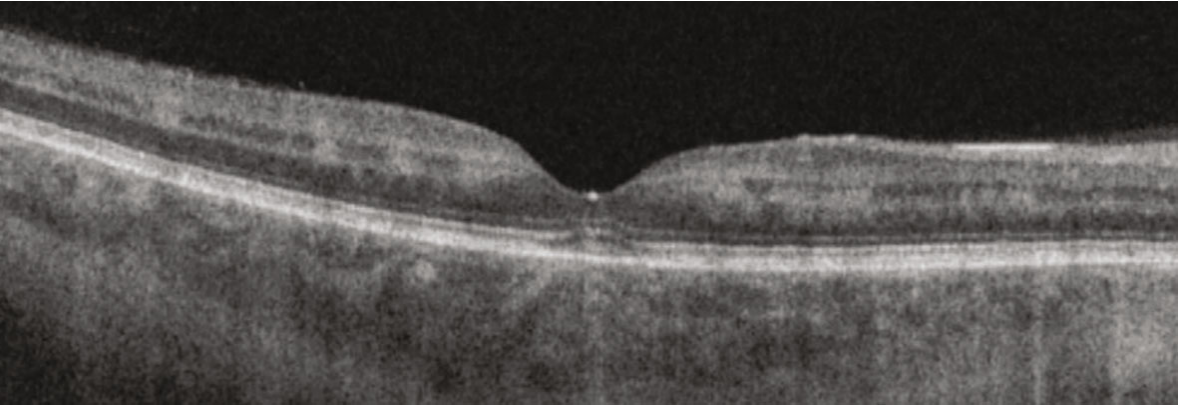
OCT B-scan at presentation demonstrating inner nuclear layer hyperreflectivity at the macula suggestive of paracentral acute middle maculopathy.

**Figure 3:**
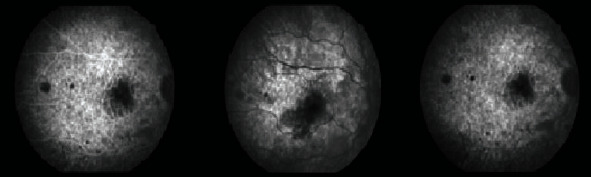
Fluorescein angiography of the right eye at presentation. One can observe the enlarged foveal avascular zone and patchy hypofluorescence in the early phases which suggest that the choroid is also involved in PLR.

**Figure 4:**
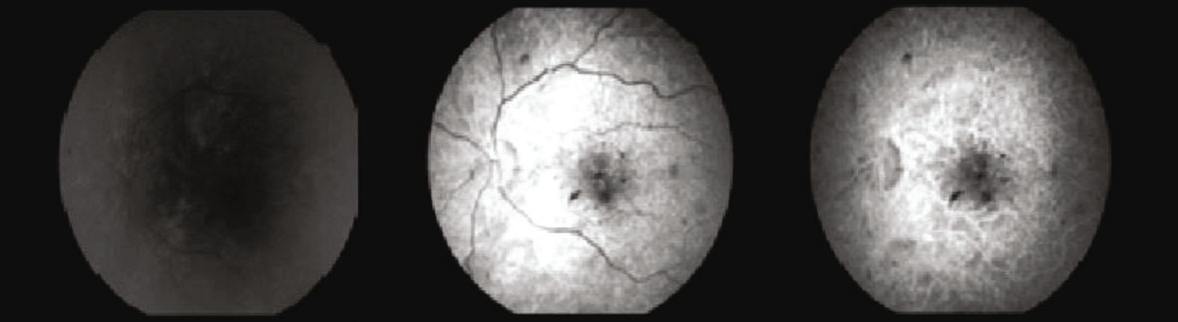
Fluorescein angiography of the left eye at presentation.

A few days after the nephrology team started their investigations, the patient presented to our eye emergency department complaining of sudden onset painless and rapidly progressive bilateral blurring of vision. Right eye visual acuity (VA) was 6/36, which improved to 6/36+1 with pinhole while the left eye VA was 6/60 and did not improve with pinhole. Pupils were both reactive with no RAPD. Ishihara colour testing was not possible as the patient could not read the test plate. Examination of the anterior segment was unremarkable. The intraocular pressures were 15 mmHg bilaterally. On dilated fundoscopy, the discs were unremarkable except for mild nasal blurring of the disc margins. Both maculae were surrounded by small patches of retinal pallor, cotton wool spots, and intraretinal haemorrhages were observed across the posterior pole and mid-periphery in both eyes (Figure 1). Optical coherence tomography (OCT) revealed bilateral hyperreflective patches at the level of the inner nuclear layer suggestive of paracentral acute middle maculopathy (Figure 2). Fluorescein angiography revealed an enlarged foveal avascular zone as well as early patchy hyperfluorescence (Figures 3 and 4). In view of a lack of history of trauma and the typical findings of perivascular patchy pallor, haemorrhages, and cotton wool spots, a diagnosis of PLR was made.

After discussion with the caring nephrology firm, it was agreed to wait for the CT-pulmonary angiogram (CTPA) before attempting a trial of steroids.

The CTPA excluded pulmonary embolism as the cause for dyspnoea and sinus tachycardia but showed faint diffuse ground glass changes which were more predominant in the perihilar areas. This was suggestive of pneumocystis pneumonia. PCR performed on bronchoalveolar lavage samples detected *Pneumocystis jirovecii* which confirmed the diagnosis of PCP. Tests for mycobacteria and galactomannan antigen were negative, and cytological analysis of the samples was unremarkable.

Given these findings, the tacrolimus dose was decreased in order to reduce the serum levels to 5-6 ng/mL, while mycophenolate was stopped temporarily. The PCP was treated with trimethoprim-sulfamethaxozole at 960 mg three times daily for a total of 21 days and then reduced to 480 mg daily indefinitely. A trial of pulsed methyl prednisolone was attempted to treat the PLR. This consisted of three doses of 250 mg IV methyl prednisolone over three days with omeprazole cover. This was followed by a course of oral prednisolone which was tapered down gradually. The patient was then left on a maintenance dose of 5 mg daily.

After commencing therapy, the dyspnoea and tachycardia resolved with PCP resolution also being confirmed on repeat CT Thorax. Patient was restarted on mycophenolate but on a lower dose of 360 mg twice daily, and the tacrolimus dose adjusted to maintain levels of around 5 ng/mL. His renal function and creatinine clearance remained stable all throughout. The patient was also seen by the dermatology team in view of the rash and was diagnosed with trichodysplasia spinulosa after histopathological analysis of a skin biopsy specimen revealed keratohyaline granules and a glass chromatic pattern. Even though viral immunostaining was negative, the patient responded well to empirical valganciclovir treatment, and the rash ultimately resolved.

**Figure 5:**
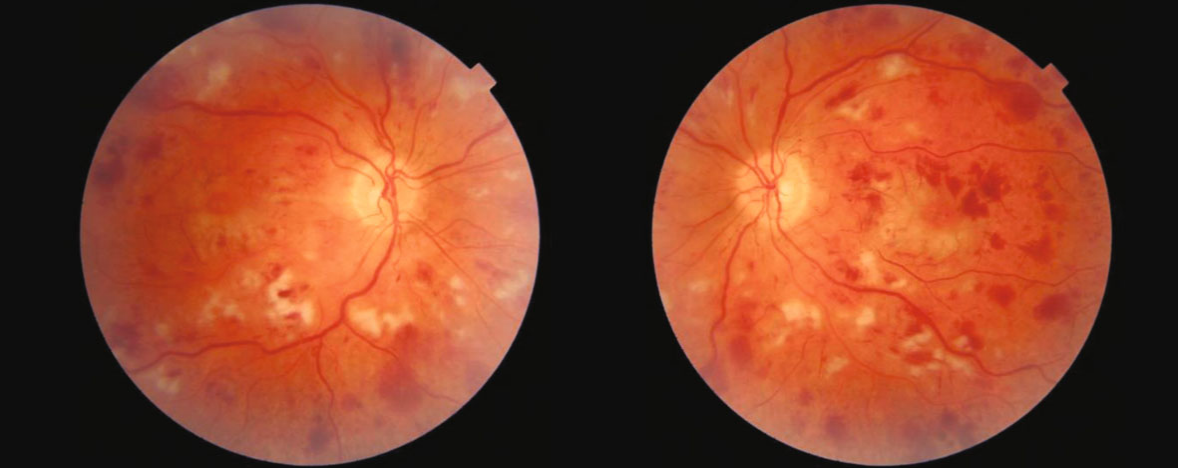
Fundal photos of the right and the left eyes 1-week postpresentation.

**Figure 6:**
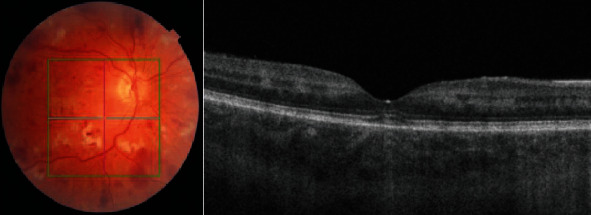
Fundal photo and OCT of the right eye 1-month postpresentation.

**Figure 7:**
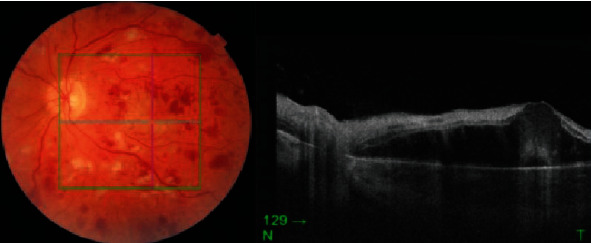
Fundal photo and OCT of the left eye 1-month postpresentation. An increase in cotton wool spots, intraretinal haemorrhages, and Purtscher's flecken was noted along with macular oedema which was confirmed on OCT.

Unfortunately, the ischaemia brought about by the PLR progressed as the patient continued losing his visual acuity. Four weeks postpresentation, the patient's VA was down to counting fingers eccentrically in both eyes. There was an increase in intraretinal haemorrhages and macular oedema on repeat fundoscopy (Figures 5–7). At that point, he was offered intravitreal bevacizumab injections but was not keen to commence such treatment. After a week, the patient accepted to undergo a trial of intravitreal bevacizumab in both eyes. The patient later developed bilateral neovascularisation at the disc (NVD) as well as a mild vitreous haemorrhage in the left eye. Bilateral pan-retinal photocoagulation was performed.

On examination, 4 weeks later, the condition was still worsening with florid NVDs present, and an epiretinal membrane had formed bilaterally. Further photocoagulation fill-in was performed.

**Figure 8:**
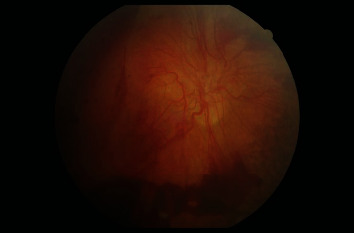
Fundal photo of the right eye 6-month postpresentation. Note: the florid NVD and inferior-lying preretinal haemorrhage.

Despite all our efforts, the VA had decreased to hand movements bilaterally at 4 months postpresentation. On fundoscopy of the right eye, one could observe a thickened posterior hyaloid and an extensive preretinal bleed which was obstructing the posterior pole view associated with a florid fibrovascular NVD (Figure 8) while in the left eye, there was a preretinal haemorrhage inferiorly as well as similar NVD plexus. A vitreoretinal opinion was obtained but due to the poor prognosis and high risk of surgery, it was agreed to proceed with further intravitreal bevacizumab injections and then consider surgery if there was no improvement.

The patient continued deteriorating with the visual field becoming more constricted and the VA decreasing to light perception in both eyes. At this point, it was agreed that surgery would not provide any benefit in view of the poor prognosis.

The patient is still being followed up in the outpatient clinic as he is at a high risk of neovascular glaucoma due to the extensive ischaemia affecting both retinas.

## 3. Discussion

To the best of our knowledge, this is the first reported case of PLR secondary to *Pneumocystis jirovecii* infection in an immunocompromised patient. PR was originally described by Otmar Purtscher after examining the fundus of a patient who had previously sustained head trauma after a fall, describing the typical signs of retinal haemorrhages and areas of retinal pallor (later coined as Purtscher's flecken) [3]. According to data collected from the British Ophthalmic Surveillance Unit, the annual incidence of symptomatic PR and PLR totals to approximately 0.24 per million per year [4].

Similar to PR, PLR is a condition with similar clinical characteristics, albeit linked to other systemic issues other than the classical association to head and thoracic trauma. The diagnostic criteria selected by Miguel et al. in their systematic review on PR and PLR include the presence of Purtscher's flecken, cotton wool spots, retinal haemorrhages, coexisting systemic inflammation or recent trauma, and hypercomplementaemia [2]. Purtscher's flecken are areas of patchy perivascular retinal pallor with a typical sparing of the immediate perivascular representing small-calibre arteriolar blockage leading to inner retinal ischaemia [1]. Cotton wool spots are manifestations of blocked axoplasmic flow secondary to ischaemia and, although not pathognomonic in themselves, they are the most frequently reported fundal finding in PR and PLR alike [2].

PLR may mimic other conditions including retinal vein and arterial occlusions (which were ruled out based on the lack of fundoscopic findings and the appearance and behaviour of the initial fluorescein angiogram) as well as HIV retinopathy and viral retinitis which is also another important aetiology in immunocompromised patients. The mode of presentation, lack of retinitis on clinical examination, and negative viral PCR results helped exclude the latter differential diagnoses.

The pathogenesis of both PR and PLR is still being debated, but bilateral ischaemic ocular changes in the light of systemic disease suggest that a widespread thromboembolic phenomenon affecting small calibre vessels, namely, precapillary arterioles, is very likely [1]. The appearance of Purtscher's flecken, characterised by retinal pallor sparing the para-arterial area, suggests that intermediate-sized emboli are most likely responsible for this phenomenon as larger emboli would obstruct the larger tributaries creating the typical branch arterial occlusion phenotype [1]. Experimental models employing the injection of graded Ballotini beads injected via the common carotid artery were performed in 1965 by Ashton and Henkind who described a similar fundal appearance due to retinal arteriole embolization [5]. It has been postulated that PLR may be related to leucoaggregation and embolization secondary to hypercomplementaemia and increased complement activation, namely, complement 5a, secondary to systemic inflammation [1] whilst PR may be related to trauma-induced vascular endothelial damage which may lead to endothelin upregulation and ultimately fibrin and platelet aggregation leading to the formation of microthrombi [6]. Literature describing cases secondary to infection is scarce, but a report by Liang and Reichel in 2015 did report complement 5a hypercomplementaemia in a patient who developed PLR secondary to a gingival abcess [7] supporting the aforementioned hypothesis. Other sources of microemboli may include fat emboli released from long bones during surgery or after sustaining fractures [8] and air embolization following trauma [9].

*Pneumocystis jirovecii* is an opportunistic fungal infection which is again on the rise in view of an increase in patients requiring chemotherapy or immunomodulatory therapy to manage cancer or autoimmune conditions or who require immune suppression following solid organ transplantation as in this case [10]. The immunopathogenesis of *Pneumocystis jirovecii* infection is still debated, but studies using animal models revealed increased pulmonary macrophage-derived complement 3 release and subsequent cleavage and activation of complement 5 [11]. This, once again, supports the hypothesis suggesting that complement-driven leucoaggregation and embolization may be the cause behind PLR secondary to systemic inflammation.

The management of PR and PLR is a matter of ongoing debate. Multiple case reports have suggested faster resolution of PR and PLR following the administration of high-dose systemic corticosteroids, but results from the systematic review performed by Miguel et al., which assimilated multiple case reports and series over the years, concluded that there was no statistically significant difference in terms of visual outcome between patients who received corticosteroid treatment and those who were observed [2]. Corticosteroids are known to affect the complement system via an upregulation of C1 inhibitors, thus arresting the complement activation cascade leading to the activation of C5 [12] which might explain how timely administration of high-dose steroids may help in the management of PLR. Further studies are required to establish their efficacy, although the paucity of PLR cases will limit study design and strength. In this case, high-dose corticosteroids were contraindicated initially in view of the fungal aetiology.

Spontaneous visual recovery, to different degrees, has been described in both PR and PLR cases with the former usually having a better outcome [2]. According to Agrawal and McKibbin, findings such as outer retinal involvement on presentation and the presence of disc leakage and choroidal hypoperfusion as well as the extent of retinal ischaemia on initial fluorescein angiography have been associated with a poorer clinical outcome [1]. Similar to this case, neovascularisation secondary to the ischaemic drive posed by PR or PLR may occur as described in a few other cases [13, 14]. Although not commonly encountered, the ischaemic pathophysiology of PR and PLR would explain why such an event may occur and supports the microembolic theory behind both variants of the disease. Despite timely management using both pan-retinal photocoagulation and bevacizumab injections, the neovascularisation encountered in this case did not regress.

In conclusion, this case provides further evidence towards the association between systemic infection and the evolution of PLR as well as highlighting the risk of developing neovascularisation in such circumstances which can, at times, be resistant to mainstay laser and intravitreal therapies.

## Data Availability

The data used to support the findings in this case report are included within the article.
